# A retrospective study on hormone replacement therapy after ovarian cancer surgery at our hospital

**DOI:** 10.20407/fmj.2024-028

**Published:** 2025-08-06

**Authors:** Arata Kobayashi, Hiroyuki Nomura, Kyohei Takada, Akiko Owaki, Mayuko Ito, Ryoko Ichikawa, Yoshiteru Noda, Hironori Miyamura, Eiji Nishio, Haruki Nishizawa

**Affiliations:** 1 Department of Obstetrics and Gynecology, Fujita Health University, School of Medicine, Toyoake, Aichi, Japan; 2 Department of Obstetrics and Gynecology, Tokai University, School of Medicine, Isehara, Kanagawa, Japan

**Keywords:** Hormone replacement therapy, Ovarian cancer, Iatrogenic menopause

## Abstract

**Objectives:**

Hormone replacement therapy (HRT) is considered for ovarian cancer patients who develop menopausal symptoms, dyslipidemia, and osteoporosis due to iatrogenic menopause caused by surgery or anticancer drug treatment. However, there have been few reports on HRT administration methods and those that evaluate cancer recurrence and complications of HRT.

**Methods:**

We examined the administration method, adverse reactions and cancer recurrence in 28 patients who received HRT after ovarian cancer surgery at our hospital.

**Results:**

All patients received estradiol monotherapy, and cancer recurrence was observed in four patients (14.3%); adverse reactions included skin eruption in two patients (7.1%), there were no other serious adverse reactions noted.

**Conclusion:**

The method and duration of HRT administration and the timing of HRT discontinuation remain debated. Thus, a large-scale survey and standardization of HRT administration methods after ovarian cancer surgery in Japan are needed.

## Introduction

According to the 2023 Vital Statistics by the Ministry of Health, Labour and Welfare, malignant neoplasms are the leading causes of death in men and women.^1^ Regarding the sites affected by cancer in women, it has been reported that breast cancer accounts for approximately 50% of cases, and gynecological cancer including ovarian cancer accounts for approximately 20%.^2,3^ In recent years, incidence of ovarian cancer has been increasing in young patients, and as the standard surgical procedure includes bilateral salpingo-oophorectomy (BSO), patients may experience menopausal symptoms associated with estrogen deficiency caused by iatrogenic menopause owing to the surgery. Additionally, dyslipidemia and osteoporosis may develop, putting patients at serious risk of cerebrovascular events and bone fractures. Specifically, it has been reported that, compared to when natural menopause occurs, when the bilateral ovaries are removed in premenopausal women, the frequency of hot flashes, a vasomotor symptom, increases significantly^4^ because of the rapid loss of estrogen secretion, and that conditions such as dyslipidemia, osteoporosis, and cardiovascular disease become more severe.^5^

To treat surgically-induced menopausal women, Japanese guidelines propose hormone replacement therapy (HRT) to maintain and improve the quality of life of patients with menopausal symptoms caused by estrogen deficiency or in patients younger than 45 years of age.^6^ However, the proactive introduction of HRT is often met with hesitation due to insufficient evidence regarding apprehension about cancer recurrence caused by the administration of hormone preparations and the onset of adverse effects associated with the administration of hormone preparations during cancer treatment.

Therefore, the present study aimed to investigate the effect of HRT on the rate of cancer recurrence and the occurrence of adverse effects by retrospectively examining patients who received HRT after ovarian cancer surgery at our hospital.

## Methods

The subject sample included 28 patients younger than 50 years of age who underwent surgical treatment including BSO for ovarian cancer at our hospital during a 5-year period from June 2019 to June 2024 and who underwent HRT after surgery. Using patient medical records, the following information was retrospectively studied: age at treatment initiation, medical history, stage and histological type of ovarian cancer, age at HRT initiation, period from the first surgery to the start of HRT, duration of HRT, administration route, symptoms and incidence of adverse reactions, details of patients with cancer recurrence and methods of evaluation after HRT administration in an outpatient setting.

Furthermore, the study was conducted with the approval of the Ethical Review Committee for Medical Research of the University (Receipt No. HM22-512).

## Results

The mean age of the patients was 40.5 years (19–49 years). Although there was no underlying illness such as diabetes or hypertension noted in the medical history, thrombosis was observed in three patients during a preoperative examination, and anticoagulation therapy was administered before HRT was introduced. The staging of ovarian cancer (FIGO 2018) was: stage I in 20 patients (stage IA in 10, stage IB in 2, and stage IC in 8), stage II in two patients (stage IIA in 1 and stage IIB in 1), stage III in five patients (stage IIIA in 2 and stage IIIB in 3), and stage IV in one patient (stage IVB in 1). The histological types of ovarian cancer were endometrial carcinoma in 4 patients, clear cell carcinoma in 6 patients, high-grade serous carcinoma in two patients, low-grade serous carcinoma in 1 patient, mucinous carcinoma in 2 patients, germ cell tumor in 3 patients, and borderline malignancy in 10 patients (Table 1). The mean age at the start of HRT was 41 years (20–50 years), the period from the first surgery to the start of HRT was 4 months (1–99 months), and the duration of HRT was 41 months (2–90 months). HRT was administered to patients by way of a patch in 18 patients (64.2%), and a cream in 10 patients (35.7%). All patients received estradiol only. HRT was discontinued or changed in seven patients, among whom two patients (7.1%) discontinued at their own request, one patient had poor compliance, and four patients requested a change to another route of administration. Adverse reactions caused by HRT included skin eruption in two patients; however, no other findings were noted (Table 2). Cancer recurrence was observed in four patients (14.2%), among whom HRT was discontinued in two patients; however, HRT was continued in the other two patients after recurrence under consultation between the attending physician and the patient (Table 3). In the evaluations performed after the start of HRT at our hospital, blood pressure, CT and tumor markers were evaluated in all 28 patients, whereas lipid parameters were evaluated in 15 patients (92.9%) and bone mineral density in 10 patients (35.7%) (Table 4).

## Discussion

In the present study, among patients who underwent surgery including BSO and HRT for ovarian cancer, number of patients with cancer recurrence was low at four patients (14.3%), and the only adverse reaction associated with HRT was skin eruption in two patients (7.1%), which was not a serious finding. Thus far, there have been no reports indicating that HRT after surgery for ovarian cancer increases the rate of cancer recurrence,^7^ which is consistent with the results of the present study.

A national population-based study in Sweden found that the majority of premenopausal women who underwent surgery for ovarian cancer did not receive postoperative HRT, indicating the need to address the use of HRT after ovarian cancer surgery in young women to prevent a decline in quality of life.^8^ However, in terms of the timing of HRT initiation, in a randomized controlled trial of HRT initiated at 6–8 weeks after surgery for 59 patients in the ET group and 66 patients in the control group who were followed-up for 4 years, it was reported that similar disease-free survival and overall survival were found.^9^ In a Swedish cohort study, 649 patients with advanced ovarian cancer enrolled in 1995 were followed-up until the end of 2002. Results revealed that patients who started HRT after completion of ovarian cancer treatment had a significantly better prognosis than those who did not receive HRT, and the results were similar for all histologic types.^10^ Reports to date have shown that HRT is not a risk factor for cancer recurrence at any stage of disease progression, and in regards to the patients from our department, HRT was administered within 3 months in 16 patients, and all patients progressed without recurrence. It has been reported that HRT increases the incidences of serous and endometrioid carcinomas in women who have not undergone surgery,^11^ and thus, a hormonal influence on the pathogenesis of ovarian cancer cannot be ruled out.

Although the 2017 edition of the Japanese guidelines for HRT states that “there is no uniform age or duration of administration that limits the continuation of HRT,” the results of a large-scale Korean study on HRT after ovarian cancer treatment revealed that the overall survival was significantly higher in the HRT-treated group. Additionally, a comparison by duration of HRT treatment showed significantly higher survival in women who received HRT for longer than 5 years.^12^ Although it is believed that HRT should be continued as long as possible in young patients, the question as to how long should it be continued for and when it should be stopped remains debated. In a recently reported subanalysis of the Women’s Health Initiative, it was reported that estrogen therapy reduces all-cause mortality in women receiving BSO at ages 50–59 years,^13^ and the question of how long HRT therapy should be continued after menopause remains open to debate.

Furthermore, there are no established test items or methods at the time of follow-up after ovarian cancer surgery with the premise of administering HRT, and according to the results of a study of Ovarian Cancer by the Tohoku Gynecologic Cancer Unit, which was reported as a large-scale survey in Japan, although tests for the purpose of cancer recurrence were conducted, tests for blood pressure and lipid abnormalities, etc. were rarely conducted.^14^ As a rule, after the start of HRT at our hospital, evaluations were performed every 6–12 months, including computed tomography and tumor markers. Blood pressure measurement were performed in all patients, liver function evaluation was performed in 93%, lipid evaluation in 54%, and bone mass measurement in 34%, all of which showed higher evaluation rates than previously reported. However, the Japanese guidelines for the treatment of ovarian cancer do not mention any specific items during HRT after ovarian cancer surgery,^6^ and these items are currently performed according to the guidelines for postmenopausal HRT, taking individual patients’ conditions into consideration. It is important to standardize the method of administering postoperative HRT, and detailed examination of follow-up for the purpose of administering HRT is required.

This was a retrospective study with a small sample size and short follow-up period. Therefore, a large-scale prospective study in Japan is needed in the future. Additionally, with recent advances in drug therapy, more patients are receiving long-term treatment for ovarian cancer with the combination of bevacizumab, olaparib, and niraparib maintenance therapy, and the relationship between new drug therapies and HRT is an issue for future study.

## Figures and Tables

**Table 1:** Patient characteristics in women with ovarian cancer <50 years at date of BSOE in author’s Hospital

Age (y)	40.5 (19–49)
BMI	21.5 (18.3–31.6)
Past history	No. (%)
VTE	3 (10.7)
Preoperative VTE	2 (7.1)
FIGO stage	No. (%)
I	20 (71.4)
II	2 (7.1)
III	5 (17.8)
IV	1 (3.6)
Subtype histology	No. (%)
EOC Endometrioid	4 (14.3)
EOC Clear cell	6 (21.4)
EOC High grade serous	2 (7.1)
EOC Low grade serous	1 (3.6)
EOC Mucinous	2 (7.1)
NEOC Germ cell	3 (10.7)
BOT	10 (35.7)

Abbreviations: VTE: venous thromboembolism; BSOE: bilateral salpingo-oophorectomy; EOC: Epithelial ovarian cancer; NEOC: Non-epithelial ovarian cancer; BOT: Borderline ovarian tumor; FIGO: International Federation of Gynecology and Obstetrics. Data are presented as n (%).

**Table 2:** HRT characteristics in women with ovarian cancer <50 years in author’s Hospital

Age of starting HRT, year	41 (20–50)
Time between primary surgery and HRT use, month	4 (1–99)
Follow-up period, month	44 (15–90)
Duration of HRT, month	41 (2–90)
Type of HRT	
Estrogen Patch, No (%)	18 (64.3)
Estrogen Liniment, No (%)	10 (35.7)
Cases of cancelling HRT, No (%)	7 (25.0)
Compliance Failure, No (%)	1 (3.6)
Desire to change to another route of administration, No (%)	4 (14.2)
Side effect of HRT	
Eruption	2 (7.1)

Abbreviations: HRT: hormone replacement therapy

**Table 3:** Cases of relapse after introduction of HRT and their background

Age	Subtype histology	FIGO 2018 stage	Time of starting HRT (Post operating month)	Duration of HRT, months	Time of recurrence (Post operating month)	Reason for suspension
46	EOC Clear cell	IC3	7	5	12	The patient’s withes
45	EOC Endometrioid	IIIC	58	14	61	Exacerbation of primary disease
34	EOC High grade serous	IIIB	36	31	56	recurrence
20	EOC Low grade serous	IIIC	1	4	16	Tumor marker rising

**Table 4:** Examinations performed in postoperative follow-up outpatient clinics for ovarian cancer in author’s Hospital

Checklists	rate of implementation (%)
Transvaginal ultrasound	64.3
Tumor marker	100
CT	100
Blood pressure measurement	100
Complete blood count assessment	92.9
Liver function assessment	92.9
Lipid assessment	53.6
Bone density assessment	35.7

## Supplementary Material

PDF-Japanese
