# Supplementary material for: A retrospective study on hormone replacement therapy after ovarian cancer surgery at our hospital
Source: Fujita Med J. 2025 Aug 6;11(4):151–4. doi: 10.20407/fmj.2024-028 (PMC12576403; doi:10.20407/fmj.2024-028)
Supplement: Supplementary file 1 — PDF-Japanese [file fmj-11-151-s001.pdf]

原著

Original Article

当院における卵巣がん術後に対するホルモン補充療法の後方視的検討

A study of hormone replacement therapy after ovarian cancer surgery at our hospital

小林新[1)], 野村弘行[1),2)], 高田恭平[1)], 大脇晶子[1)], 伊藤真友子[1)], 市川亮子[1)], 野田佳照[1)], 宮村浩徳[1)], 西尾永司[1)], 西澤春紀[1)]

Arata Kobayashi M.D., Hiroyuki Nomura Ph.D., Kyohei Takada M.D., Akiko Owaki Ph.D., Mayuko Ito Ph.D., Ryoko Ichikawa M.D., Yoshiteru Noda Ph.D., Hironori Miyamura Ph.D., Eiji Nishio Ph.D., Haruki Nishizawa Ph.D.

[1)]藤田医科大学医学部産婦人科

Department of Obstetrics and Gynecology, Fujita Health University, School of Medicine, Toyoake city, Aichi prefecture, Japan

[2)]東海大学医学部産婦人科

Department of Obstetrics and Gynecology, Tokai University School of Medicine, Isehara city, Kanagawa prefecture, Japan

ランニングタイトル: HRT after ovarian cancer surgery

Corresponding author:

Arata Kobayashi M.D.

Department of Obstetrics and Gynecology, Fujita Health University, School of Medicine,

1-98, Dengakugakubo, Kutsukake-cho, Toyoake, Aichi 470-1191

Tel: 0562-93-9294; Fax: 0562-95-1821

E-mail: arata.kobayashi@fujita-hu.ac.jp

［抄録］
【目的】卵巣がんは，手術や抗がん剤治療により医原性閉経になることで，更年期症状をはじめ，脂質異常症や骨粗鬆症を発症するため，ホルモン補充療法が考慮されるが，投与法と投与後のがん再発や合併症などの評価をしている報告はまだ少ない.【方法】当院における卵巣癌の術後に HRT を施行した 28 症例を対象として，投与法と副作用，またがん再発について検討した.【結果】全例でエストラジール製剤の単独投与が行われ，がんの再発は 4 例（14.3%）に認め，副作用は皮疹を 2 例（7.1％）に生じたが重篤な副作用は認めなかった.【結論】HRT の投与方法や期間，また中止時期についてのコンセンサスは得られていないため，国内における大規模な調査と卵巣がん術後の HRT 投与法の標準化が望まれる.

Key word; Hormone Replacement Therapy, Ovarian Cancer, Iatrogenic menopause

［緒言］

厚生労働省による2023年の人口動態統計によれば，男女ともに死因第1位は悪性新生物であり[1]，女性における癌の罹患部位としては，乳癌が約50%を占め，卵巣癌を含めた婦人科がんは約20%を占めると報告されている[2,3]．近年，卵巣癌は若年者で増加傾向にあり，両側付属器摘出術（bilateral salpingo-oophorectomy；BSO）が標準術式に含まれるため，手術の実施により医原性閉経となることで，エストロゲン欠落に伴う更年期症状を呈するとともに，脂質異常症や骨粗鬆症を発症し，脳・心血管系イベントや骨折等の重篤なリスクを生じる.特に，閉経前女性に対して両側卵巣を摘出した場合，自然閉経で起こる場合に比べてエストロゲン分泌が急激に消失するため，血管運動神経症状であるホットフラッシュの出現頻度が有意に増加し[4]，脂質異常症，骨粗鬆症，心血管系疾患などの各病態が重症化することも報告されている[5]．

一方，こうした外科的閉経女性への対応として，国内のガイドラインではエストロゲン欠落に伴う更年期症状を有する場合や45歳未満の場合にはQOL維持改善のためにホルモン補充療法（Hormone replacement therapy：HRT）が提唱されているが[6]，ホルモン剤の投与によるがん再発への懸念や，がん治療中のホルモン剤投与にともなう副障害の発生等について十分なエビデンスが得られているとは言えないため，積極的なHRTの導入に躊躇することも少なくなかった.

そこで，本研究では，当院における卵巣がん術後に対するHRT投与例を後方視的に調査し，がん再発率への影響や副障害の発生について検討することを目的とした.

［方法］

2019年6月～2024年6月の5年間に，当院にて卵巣癌に対してBSOを含む手術療法を施行した症例で，術後にHRTを施行した50歳未満の28症例を対象とした. 診療録より，治療開始年齢，既往歴，卵巣がん進行期と組織型，HRT開始年齢，初回手術からHRT開始までの期間，HRT施行期間，投与経路，副作用の症状と発現率，がん再発例の詳細および外来でのHRT投与後の評価方法について後方視的に

調査した．なお，本研究は当大学医学研究倫理審査委員会において承認を得たうえで実施した（受付番号 HM22-512）．

［結果］

対象者の年齢は 40.5 歳(19-49 歳)，BMI，既往歴では糖尿病，高血圧症等の基礎疾患は認めなかったが，術前検査により血栓症を 3 例に認め，HRT 導入前に抗凝固療法が施行された.卵巣がんの進行期分類(FIGO 2018)は，I期 20 例(IA 期 10 例，IB 期 2 例，IC 期 8 例)，II期 2 例(IIA 期 1 例，IIB 期 1 例)，III期 5 例(IIIA 期 2 例，IIIB 期 3 例)，IV期 1 例(IVB 期 1 例)であった．また，卵巣がんの組織型は類内膜癌 4 例，明細胞癌 6 例，高異型度漿液性癌 2 例，低異型度漿液性癌 1 例，粘液性癌 2 例，胚細胞腫瘍 3 例，境界悪性腫瘍は 10 例であった (Table. 1) .

次に HRT 開始年齢は 41 歳(20-50 歳)で，初回手術から HRT 開始までの期間は 4 カ月(1-99 カ月)，HRT 施行期間は 41 ヶ月(2-90 カ月)であった．HRT の種類は貼付剤が 18 例(64.2%)，塗布剤が 10 例（35.7%）で，全例エストラジール製剤のみが投与されていた．HRT の中止または変更を 7 例に認め，そのうち本人希望での中止が 2 例(7.1%)，コンプライアンス不良が 1 例，他投与経路への変更希望が 4 例であった．

HRT による副作用は，皮疹を 2 例に生じたが，他の所見は認めなかった（Table. 2）．

がんの再発は 4 例（14.2%）に認め，そのうち 2 例で HRT 投与が中止されていたが，他の 2 例については再発後も主治医と患者の協議の下で HRT が継続されていた（Table. 3）.当院における HRT 開始後の評価については，血圧，CT，腫瘍マーカーは 28 例と全例で施行されていたが，脂質項目は 15 例(92.9%)，骨密度は 10 例(35.7%)に実施されている状況であった（Table. 4）．

［考察］

本研究において，卵巣癌に対して BSO を含む手術療法ならびに HRT 施行例のうち，がん再発例は 4 例(14.3%)と低く，また HRT 投与に伴う副作用は，皮疹を 2 例(7.1%)に認めたのみであり，重篤な所見は認めていない.これまで，卵巣癌術後の HRT により再発率が上昇するという報告はみられず [7]，本研究でも概ね矛盾しない結

果を得られた.スウェーデンの全国集団ベース研究では，卵巣癌術後の閉経前女性の大多数は術後に HRT を施行していなかったことから，生活の質の低下を防ぐために，若い女性における卵巣癌術後の HRT 使用に取り組む必要性があることを示している [8]．一方，HRT の開始時期における検討では，術後 6〜8 週間で HRT を開始した Randomized Controlled Trial(RCT)の報告があり，ET 群 59 例，対照群 66 例を 4 年間フォローした結果，無病生存期間も全生存期間も同等であった [9]．また，スウェーデンのコホート研究では，1995 年に登録した 649 例の進行卵巣癌患者を 2002 年末まで追跡した結果，卵巣癌治療終了後に HRT を開始した患者群の方が HRT 無施行群に比べ有意に予後が良好であり，この結果は全組織型で同様な結果であった [10]．従って，進行期はいずれにおいても HRT が再発のリスク因子とならないことがこれまでの報告より示されており，当科の症例においても，16 例で 3 ヶ月以内に HRT を施行し，全例再発なく経過している．一方で、手術既往のない女性にホルモン補充療法を行うことにより漿液性癌と類内膜癌の発生が増加することが報告されているため [11]、卵巣癌の発生機序にホルモンが影響することは否定できない。

国内のホルモン補充療法ガイドライン 2017 年度版において「HRT の継続を制限する一律の年齢や投与期間はない」とされているが，韓国の卵巣癌治療後の HRT 施行に関する大規模な研究結果では，全生存率は HRT 施行群で有意に高く，さらに HRT 施行期間による比較では 5 年以上の長期間行った女性で有意に生存率が高かったことが示された [12]．若年の場合は可能な限り HRT は継続するべきであるという意見がある一方，いつまで続けるか，いつ中止するかについては統一した見解が得られていないのが現状である．最近報告された Woman’s Heawlth Initiative (WHI)研究のサブ解析結果において，50~59 歳で BSO を受けた女性において，エストロゲンの投与により全死亡率が低下することも報告されていることより [13]，今後，閉経年齢以降いつまで継続するかについては議論の余地がある.

さらに，卵巣癌術後のフォローアップ時に，HRT 投与を前提とした検査項目やその方法に確立されたものはなく，国内の大規模な調査として報告された卵巣がん東北癌化学療法研究会の結果では，がん再発を目的とした検査は行われているものの，血圧や脂質異常等に対する検査は，ほとんど行われていなかった [14]．当院における

HRT 開始後の評価は 6〜12 ヶ月毎に行うことを原則とし，CT，腫瘍マーカーを含め，血圧測定は全例に対して実施され，肝機能評価は 93%，脂質評価は 54%，骨量測定は 34%に施行されており，いずれも既報告より評価率は高かった.その一方で，国内の卵巣がん治療ガイドラインでは卵巣癌術後に特記した HRT 中の実施項目についての言及はなく[6]，閉経後のホルモン補充療法のガイドラインに準じて個々の患者の状態を勘案しながら施行するのが現状となっている. 術後 HRT 投与については，その方法について標準化することが重要であり，HRT 投与を目的としたフォローアップについても詳細な検討が必要と思われた.

本研究は，サンプル数少数の後方視的研究であり，追跡機関が短い等の問題があるため，今後は，国内における大規模な前向き研究が望まれる.また，近年は薬物療法の進歩により，ベバシズマブ，オラパリブ，ニラパリブの併用維持療法により卵巣癌の治療が長期におよぶ症例が増えているため，新規薬物療法と HRT の関連についても今後の検討課題である.

［利益相反］

この論文に関連して開示すべき利益相反状態にはありません.

［謝辞］

本論文を執筆するにあたり指導してくださった医局員の方々に感謝の意を表する.

［引用文献］

1) Ministry of Health, Labour and Welfare. 2023 Population Statistics (Final Data) Overview;2024（in Japanese）. <https://www.mhlw.go.jp/toukei/saikin/hw/jinkou/geppo/nengai23/dl/gaikyouR5.pdf>(Accessed September 18, 2024)

2) National Cancer Center. Cancer Statistics; 2023.（in Japanese） <https://ganjoho.jp/public/qa_links/report/ statistics/2023_jp.html>(Accessed September 18, 2024)

3) Ogawa M, Shiraishi M, Takamatsu K. Fujinka gan sabaiba heno hormone hoju ryoho. Sanfujinka no jissai 2023;72: 1015-21（in Japanese）.

4) Gallicchio L , Whiteman MK, Tomic D, Miller KP, Langenberg P, Flaws JA. Type of menopause, patterns of hormone therapy use, and hot flashes. Fertil Steril 2006; 85: 1432-40.

5) Singh P, Oehler MK. Hormone replacement after gynaecological cancer. Maturitas 2010; 65: 190-7.

6) Japan Society of Gynecologic Oncology. Guidelines for treatment of ovarian cancer, fallopian tube cancer and primary peritoneal cancer. 2020 ed. Tokyo: Kanehara Shuppan; 2020: 128-30（in Japanese）.

7) The NAMS 2017 Hormone Therapy Position Statement Advisory Panel. The 2017 hormone therapy position statement of The North American Menopause Society. Menopause 2017; 24:728-53.

8) von Kartaschew ÅE, Dahm-Kähler P, Rodriguez-Wallberg KA, Holmberg E, Rådestad AF. Hormone replacement in premenopausal women treated with bilateral oophorectomy for ovarian cancer - a nationwide population-based study. Gynecol Oncol 2022;167:476-82.

9) Guidozzi F, Daponte A. Estrogen replacement therapy for ovarian carcinoma survivors: A randomized controlled trial. Cancer 1999;86:1013-8.

10) Mascarenhas C, Lambe M, Bellocco R, Bergfeldt K, Riman T, Persson I, Weiderpass E. Use of hormone replacement therapy before and after ovarian cancer diagnosis and ovarian cancer survival. Int J Cancer 2006;119:2907-15.

11) Beral V, Gaitskell K. Menopausal hormone use and ovarian cancer risk: individual

participant meta-analysis of 52 epidemiolog- ical studies. Lancet. 2015; 385: 1835-42.

12) Ji E, Kim K, Lee B, Hwang SO, Lee HJ, Lee K, Lee M, Kim YB. Postoperative hormone replacement therapy and survival in women with ovarian cancer. Cancers (Basel) 2022;14:3090.

13) Manson JE, Aragaki AK, Bassuk SS, et al. Menopausal estrogen- alone therapy and health outcomes in women with and without bilateral oophorec- tomy: a randomized trial. Ann Intern Med 2019;171:406-14.

14) Mizunuma H. Joseiigaku-Aratana sanfujinka senmonryoiki no setsuritsu to mokuteki-. Sanka to Fujinka 2011;78:1445-50（in Japanese）.

Table 1. Patient characteristics in women with ovarian cancer <50 years at date of BSOE in auther's Hospital.

Age (y)	40.5 (19-49)
BMI	21.5 (18.3-31.6)
Past history	No. (%)
VTE	3 (10.7)
Preoperative VTE	2 (7.1)
FIGO stage	No. (%)
I	20 (71.4)
II	2 (7.1)
III	5 (17.8)
IV	1 (3.6)
Subtype histology	No. (%)
EOC Endometrioid	4 (14.3)
EOC Clear cell	6 (21.4)
EOC High grade serous	2 (7.1)
EOC Low grade serous	1 (3.6)
EOC Mucinous	2 (7.1)
NEOC Germ cell	3 (10.7)
BOT	10 (35.7)

Abbreviations: VTE: venous thromboembolism; BSOE: bilateral salpingo-oophorectomy; EOC: Epithelial ovarian cancer; NEOC: Non-epithelial ovarian cancer; BOT: Borderline ovarian tumor; FIGO: International Federation of Gynecology and Obstetrics. Data are presented as n (%).

Table 2. HRT characteristics in women with ovarian cancer <50 years in auther's Hospital.

Age of starting HRT, year	41 (20-50)
Time between primary surgery and HRT use, month	4 (1-99)
Follow-up period, month	44 (15-90)
Duration of HRT, month	41 (2-90)
Type of HRT	
Estrogen Patch, No (%)	18 (64.3)
Estrogen Liniment, No (%)	10 (35.7)
Cases of cancelling HRT, No (%)	7 (25.0)
Compliance Failure, No (%)	1 (3.6)
Desire to change to another route of administration, No (%)	4 (14.2)
Side effect of HRT	
Eruption	2 (7.1)

Abbreviations: HRT: hormone replacement therapy

Table 3. Cases of relapse after introduction of HRT and their background.

Age	Subtype histology	FIGO 2018 stage	Time of starting HRT (Post operating month)	Duration of HRT, months	Time of recurrence (Post operating month)	Reason for suspension
46	EOC Clear cell	IC3	7	5	12	The patient's withes
45	EOC Endometrioid	IIIC	58	14	61	Exacerbation of primary disease
34	EOC High grade serous	IIIB	36	31	56	recurrence
20	EOC Low grade serous	IIIC	1	4	16	Tumor marker rising

Table 4. Examinations performed in postoperative follow-up outpatient clinics for ovarian cancer in auther's Hospital.

Checklists	rate of implementation(%)
Transvaginal ultrasound	64.3
Tumor marker	100
CT	100
Blood pressure measurement	100
Complete blood count assessment	92.9
Liver function assessment	92.9
Lipid assessment	53.6
Bone density assessment	35.7
